# Risk factors of mild rectal bleeding in very low birth weight infants: a case control study

**DOI:** 10.1186/1471-2431-13-196

**Published:** 2013-11-27

**Authors:** Abdallah Oulmaati, Stephane Hays, Mohamed Ben Said, Delphine Maucort-Boulch, Isabelle Jordan, Jean-Charles Picaud

**Affiliations:** 1 Department of Neonatology, Hopital de la Croix Rousse, Hospices Civils de Lyon, F-69004, Lyon, France; 2 Rhone-Alpes Human Nutrition Research Center, Centre hospitalier Lyon Sud, Pierre-Benite F-69310, France; 3 Department of Biostatistics, Hospices Civils de Lyon, F-69003, Lyon, France; 4 CNRS, Laboratoire Biostatistique Sante, UMR 5558, Pierre Benite F-69310, France; 5 Faculte de Medecine Lyon-Sud Charles Merieux, Universite Claude Bernard Lyon 1, F-69100, Villeurbanne, France

**Keywords:** Prematurity, Necrotising enterocolitis, Nonsteroidal anti-inflammatory, Hypertension, Nutrition

## Abstract

**Background:**

Mild rectal bleeding (MRB) is a particular clinical entity different from necrotizing enterocolitis, which significantly influences neonatal care in preterm infants. We aimed to determine the risk factors and to evaluate prospectively the clinical course of MRB.

**Methods:**

We consecutively included in a case–control study all infants with birth weight ≤ 1500 g or gestational age ≤ 32 weeks admitted to our unit, and presenting MRB, defined as either isolated or associated with mild clinical or radiological signs. We matched each Case with two Controls. Clinical data before, after and at time of MRB were collected, together with stool cultures at time of MRB (or at similar postnatal age in Controls). Multiple logistic regression analysis was performed to determine independent risk factors for the development of MRB.

**Results:**

During 4 years, among 823 very low birth weight (VLBW) infants admitted to our unit, 72 (8.8%) had MRB. The median duration of rectal bleeding was 1.1 [1–2] days and the fasting period lasted 2.9 [2–10] days. A relapse occurred in 24% of cases. In multivariate analysis, only hypertension during pregnancy (p = 0.019), growth restriction at onset of bleeding (p = 0.026), and exposure to ibuprofen (p = 0.003) were independent risk factors for MRB. In Cases there were more infants with *Clostridium Difficile* in stools than in Controls (p = 0.017).

**Conclusion:**

Hypertension during pregnancy, even without intrauterine growth restriction, appeared to carry the same risk for MRB as exposure to ibuprofen and extrauterine growth restriction.

## Background

Intestinal immaturity in very low birth weight (VLBW) infants causes digestive disorders. It ranges from feeding intolerance (gastric residuals, abdominal distention, etc.) to necrotising enterocolitis (NEC) which involves an inflammatory intestinal condition [1,2]. In preterm infants, rectal bleeding may be isolated or not. When it is associated with mild clinical or radiological signs, it is classified as “suspected NEC” or NEC stage 1B [3]. According to the classification proposed by Vermont Oxford Network, clinical (including occult or gross blood in stools) and radiographic findings, with one or more of each (clinical or radiographic) is required for a diagnosis of NEC [4]. Therefore, none of these two classifications takes in account isolated rectal bleeding. While there is a lot of publications about NEC (stage ≥ 2), published data about isolated rectal bleeding in VLBW infants are scarce [5,6].

In daily practice rectal bleeding, that is either completely isolated or associated with mild clinical symptoms or radiological signs, is quite frequent. It is a particular entity which could be grouped under the name of mild rectal bleeding (MRB).

However, there is still no recommendation about prevention of MRB in VLBW infants, probably because little is known about risk factors of MRB. This is unfortunate as it may have a significant impact on neonatal care because the management sometimes requires a fasting period [3], with possible need to start or extend parenteral nutrition, which increases the risk of catheter-related sepsis [7,8].

We aimed to evaluate the incidence and determine the risk factors of mild rectal bleeding in very low birth weight infants.

## Methods

### Study design

This was a single centre retrospective case–control study.

### Population

All children with birth weight (BW) ≤ 1500 g or gestational age (GA) ≤ 32 weeks admitted to the tertiary neonatal intensive care unit of the University Hospital of the Croix Rousse in Lyon were eligible for the study.

The Cases were preterm infants who had MRB; that is to say, either isolated or with mild clinical (mild abdominal distention, elevated pre-gavage residuals or emesis) or radiological (intestinal dilatation, mild ileus) signs, different from NEC stage 2 which is characterized by specific radiological signs such as pneumatosis intestinalis [3]. When a patient had multiple incidents of rectal bleeding, we included only the first episode. When a relapse took place more than 7 days after rectal bleeding, it was considered as a recurrence. Otherwise it was considered as related to the first event.

We matched each Case with two Controls (one of each gender). Controls were the first infants hospitalized in our unit to be born after a corresponding Case with the same body weight and gestational age (GA) at birth, respectively ±100 g and ±1 week.

We excluded from the study children with anal fissures, NEC ≥ stage II, or spontaneous intestinal perforation, as well as children who had a foetopathy, genetic disorders or severe malformations. Swallowing blood syndrome can be ruled out in the preterm group, as feeding is by either bottle or nasogastric tube. For the Controls, we excluded infants who died before the postnatal age of onset of rectal bleeding in the corresponding Case.

Feeding regimen of the VLBW infants was not changed during the study period: enteral feeding was started at day 1 or 2 and complementary parenteral feeding was administered until enteral intake reached 100 ml/kg/day. All infants were fed pasteurized human milk (own mother’s milk or donor milk) until body weight was around 1500 g. Then, if the mother had no milk, a preterm formula was proposed. Human milk (either pasteurized own mother’s milk or pasteurized donor milk) was supplemented using a multicomponent fortifier (Eoprotine, Milupa) as soon as the enteral intake reached 100 mL/kg/day). We added 4 g of powder per mL, which induces a significant energy and protein fortification up to 80 kcal and 2.2 g/100 mL respectively. Full enteral feeding was 160 ml/kg/day and was reached by increasing by 10 to 20 ml/kg/d depending on feeding tolerance. After full enteral feeding (160 ml/kg/day) has been reached, when infants presented signs of gastro-oesophageal reflux we used a thickener (starch-based when fed human milk, carob-based when fed a preterm formula).

Mild rectal bleeding may be isolated or not. When it is associated with mild clinical or radiological signs, it is classified as “suspected NEC” or NEC stage 1B [3]. According to our protocol, infants with rectal bleeding went through a fasting period and were fed only parenterally. The fasting period lasted between 1 and 10 days depending on the duration of rectal bleeding and the clinical (mild abdominal distention, elevated pre-gavage residuals or emesis) or radiological (intestinal dilation, mild ileus) signs associated with the bleeding. Wide spectrum antibiotics were prescribed when there were associated clinical or radiological signs.

In addition, we collected data from systematic weekly stool cultures routinely performed in all VLBW infants to monitor for the emergence of multi-resistant bacteria.

### Data collection

We collected antenatal data (maternal illness, medications during pregnancy, mode of delivery), infant characteristics at birth (Apgar score, GA and birth weight). Infants were considered growth restricted when birth weight was less than −2 standard deviations (SD) [9]. We collected history before the occurrence of rectal bleeding: drugs, ventilatory support, oxygen therapy, feeding, and proportion of infants who received thickeners.

At the time of rectal bleeding (or an equivalent postnatal age in Controls), we collected postnatal age, duration of rectal bleeding, treatment, and type of enteral feeding. For each Control, we collected the characteristics between birth and the postnatal age at the onset of rectal bleeding in the corresponding Case.

We collected the results of the stool cultures performed for identification of pathogens (*Staphylococcus aureus, Coagulase negative Staphylococcus, Clostridium difficile, Escherichia coli, Enterococcus, Klebsiella pneumoniae, Proteus mirabilis, Proteus mirabilis, Klebsiella oxytoca*) in the week before the occurrence of rectal bleeding in Cases and at equivalent GA and postnatal age in Controls.

### Statistical analysis

Results are expressed as numbers (percentage) or medians [min; max]. Case and Control groups were compared using the Wilcoxon test for quantitative parameters and the CHI-2 or Fisher’s exact test when appropriate for categorical variables. All parameters with a p value below 0.1 were stepwise introduced into a multiple logistic regression where being a Case or a Control was the dependent variable. We used SPSS 16 Software (SPSS Inc., Chicago, IL) to perform statistical analysis.

### Ethics

The study protocol was approved by the ethics committee of Lyon (*Comité de protection des personnes Sud Est IV Lyon*).

## Results

Between January 2007 and December 2010, 823 children with a birth weight ≤ 1500 g or GA ≤ 32 weeks were admitted consecutively to the neonatal unit of Croix Rousse University Hospital. Of these, 75 had MRB. Three children were excluded. The first one had a foetopathy due to cytomegalovirus. The second died at day 67 (corrected GA: 35 weeks) from intestinal perforation and sepsis. The last had a severe malformation (chylothorax and chylous ascites). We then analysed the data in the 72 infants corresponding to the inclusion criteria, which was 8.8% (72/820) of the population.

Among the 72 VLBW infants included in our study, over one third (28/72, 39%) had isolated rectal bleeding and the others (44/72, 61%) had rectal bleeding associated with mild clinical or radiological signs. It represented respectively 3.4% and 5.4% of the whole population of VLBW infants.

The rectal bleeding occurred at a median postnatal age of 28 [7–108] days and lasted 1.1 [1,2] days. Management consisted of a fasting period lasting 2.9 [2–10] days. A relapse occurred in about one case out of four (17/72, 24%), and it was 17 [10–50] days after the first episode. In most of the cases (16/17, 94%) the recurrence was a MRB, isolated in almost all cases (15/16), only 1 being associated with clinical or radiological signs. In 1 case, the relapse was not a MRB but a NEC (stage II) that occurred 15 days after the initial rectal bleeding.

Of the 748 children who showed no rectal bleeding, we selected 144 Controls meeting the inclusion criteria. The characteristics of the mothers of Cases and Controls were similar during pregnancy and childbirth apart from hypertension, which was significantly more frequent in mothers of Cases (Table 1). The characteristics of the infants at birth were similar in the two groups (Table 1). Enteral feeding was started in the 1st day of life in most infants and there was no significant difference between Controls (90.3%) and Cases (93.1%) (p = 0.439).

**Table 1:** Clinical characteristics in 216 preterm infants with (Cases) or without (Controls) rectal bleeding

	**Controls n = 144**	**Cases n = 72**	p*
**Pregnancy and delivery**			
Singleton, n (%)	99 (68.8)	49 (68.05)	0.835
Antenatal steroids, n (%)	123 (85.4)	64 (88.9)	0.876
Hypertension, n (%)	14 (9.7)	16 (22.2)	0.03
Diabetes, n (%)	10 (6.9)	11 (15.3)	0.168
Cesarean section, n (%)	102 (73.6)	48 (66.7)	0.250
**At birth**			
Birth weight (g)	1150 [500–1495]	1140 [540–1480]	0.831
Gestational age (weeks)	29 [24–32]	28 [24–32]	0.446
Birth weight < −2SD, n (%)	30 (20.8)	16 (22.2)	0.814
**Drugs before rectal bleeding**			
Antiacids, n (%)	19 (13.1)	12 (16.7)	0.663
Ibuprofen, n (%)	19 (13.2)	22 (30.6)	0.003
Postnatal steroids, n (%)	4 (2.8)	6 (8.3)	0 .087
**Respiratory support before rectal bleeding**			
Assisted ventilation (hours)	19 [0–1916]	23.5 [0–1311]	0.955
CPAP (hours)	124 [0–2411]	212 [0–9312]	0.178
Oxygen therapy (hours)	15 [0–2913]	39 [0–2720]	0.567

Number of subjects (%) or median [min-max].

*Chi-2 test (or Fisher’s exact test when required) and Wilcoxon test were performed to compare qualitative and quantitative value, respectively.

There was no difference between the two groups of infants before the onset of rectal bleeding, except for the proportion of subjects treated with ibuprofen (p = 0.003) (Table 1). The proportion of children postnatally treated with steroids tended to be higher in Cases, but it did not reach significance (p = 0.087).

At the time of rectal bleeding, there was a tendency for a higher proportion of subjects with weight below -2SD among the Cases (p = 0.08), but there were no other significant differences between the groups, including respiratory support. All infants were exclusively on enteral feeding and most infants were fed pasteurized human milk (own mother’s milk or donor milk), without significant difference between the two groups (Table 2).

**Table 2:** Characteristics of 216 preterm infants at time of rectal bleeding in cases and before the corresponding postnatal age in controls

	**Controls n = 144**	**Cases n = 72**	p*
**Characteristics at time of rectal bleeding**			
Postconceptional age (weeks)	34 [30–42]	34 [28–45]	0.1
Body weight (g)	1829 [1138–5030]	1690 [940–4630]	0.230
Body weight < −2 SD, n (%)	51 (35.4)	34 (47.9)	0.08
X-ray intestinal distension, n (%)	12 [8;3]	29 [40;3]	0.001
**Enteral feeding at time of rectal bleeding**			
Human milk, n (%)	61 (42.4)	48 (66.7)	0.439
Preterm formula, n (%)	52 (36.1)	15 (20.8)	0.136
Human milk and preterm formula n, (%)	31 (21.5)	9 (12.5)	0.425
Starch-based thickener, n (%)	13 (9)	4 (5.6)	0.462
Carob-based thickener, n (%)	8 (5.6)	1 (1.4)	0.558

Number of subjects (%) or median [min-max].

*Chi-2 test (or Fisher’s exact test when required) and Wilcoxon test were performed to compare qualitative and quantitative value, respectively.

In addition to the two parameters significantly associated with the occurrence of rectal bleeding in univariate analysis (hypertension during pregnancy, exposure to ibuprofen), postnatal exposure to steroids and body weight < −2SD at time of rectal bleeding were introduced in a stepwise multivariate model to determine the independent risk factors for MRB (Table 3). Both hypertension and ibuprofen treatment remained significant risk factors, and low body weight for corrected gestational age at the onset of bleeding became significant. In summary, hypertension during pregnancy (p = 0.019), exposure to ibuprofen (p = 0.003) and being growth restricted at onset of rectal bleeding (p = 0.026), were independent risk factors for MRB in the study population. In other words, VLBW infants of mothers who presented hypertension during pregnancy, those who received Ibuprofen, or those who poorly grew postnatally had a 2 to 3 times greater risk of having MRB.

**Table 3:** Risk factors for occurrence of rectal bleeding in 216 preterm infants, results from multivariate analysis

	**OR**	**[95% CI]**	**p**
Ibuprofen	3.193	[1.470-6.937]	0.003
Postnatal steroid exposure	2.839	[0.584-11.784]	0.151
Hypertension during pregnancy	2.607	[1.174-5.787]	0.019
Body weight at bleeding < −2SD	2.029	[1.088-3.783]	0.026

SD: standard deviation, OR: odds ratio, [95% CI]: 95% confidence interval.

The proportion of infants with pathogens in stools was similar in the two groups (Table 4). About one in three infants had stool culture positive for *Staphylococcus*, while *Clostridium difficile* was rarely found. In Cases, proportion of infants with *Clostridium Difficile* was higher than in Controls (p = 0.017) while there was significantly more infants with more than 2 pathogens isolated amongn Controls (p < 0.001).

**Table 4:** Results of stool cultures performed during the week before the occurrence of rectal bleeding in cases and during the week before the corresponding age in controls

	**Controls n = 144**	**Cases n = 72**	p*
Positive stool culture, n (%)	93 (64)	39 (54)	0.139
*Staphylococcus aureus*, n (%)	18 (12.5)	13 (18.1)	0.272
*Coagulase negative Staphylococcus*, n (%)	31 (21.5)	13 (18.1)	0.55
*Clostridium difficile*, n (%)	1 (0.7)	5 (6.9)	0.017
*Escherichia coli*, n (%)	6 (4.2)	2 (2.8)	0.722
*Enterococcus*, n (%)	9 (6.3)	2 (2.8)	0.343
*Klebsiella pneumoniae*, n (%)	2 (1.4)	4 (5.6)	0.097
*Proteus mirabilis,* n (%)	1 (0.7)	0	1
*Klebsiella oxytoca,* n (%)	1 (0.7)	0	1
More than two micro organisms, n (%)	24 (16.7)	0	< 0.001

Results expressed as number of subjects (%).

*Chi-2 test or Fisher’s exact test when required.

## Discussion

In our population of VLBW infants, we found that maternal hypertension during pregnancy, postnatal growth restriction, and treatment with ibuprofen were independent risk factors for the occurrence of rectal bleeding associated or not with mild clinical or radiological signs (mild rectal bleeding).

Nearly one tenth of these preterm infants presented MRB. While we have data about the prevalence of isolated rectal bleeding (2.3%) and NEC ≥ stage 2 (3 to 8% depending on gestational age at birth) [2,6,10], to our knowledge it is the first report of the prevalence of MRB, as defined here, in VLBW infants.

When considering only isolated rectal bleeding, we observed a prevalence of 3.4% in our VLBW infants, which is close to what has been reported by Maayan-Metzger et al. (3.7%) in preterm infants ≤ 34 weeks [6].

The overall rate of MRB was higher in our study (8.8%) than in previous studies, because we also considered rectal bleeding with mild clinical and radiological signs, which is clinically relevant because it is a common clinical situation requiring similar care in daily practice.

Relapses was slightly more frequent (24%) to what has previously been reported (17%) [6]. That difference could be related to the greater immaturity in our population of VLBW infants than in previous study [6]. However recurrences are mainly isolated rectal bleeding, suggesting that MRB could not be considered as a risk factor for later NEC, but it has to be confirmed in larger populations.

Although our study was retrospective, each Case was carefully matched with two Controls, which was effective as the two groups were quite comparable at the time of rectal bleeding. Contrary to what has been performed by Maayan-Metzger et al. [5] who paired only one Controls with each case. As Controls were the first infants hospitalised in our unit to be born after a corresponding Case, they were hospitalized at the same period than Cases, i.e. exposed to similar care (feeding protocol, antibiotics), in the same environment, which is relevant as we collected information about care, presence of pathogens in stools and clinical evolution. Although the monocentric character is an obvious limitation, this single-centre study with a protocol for managing rectal bleeding and carried out over a relatively short period, had limited or no bias associated with differences in practices between centres or changes in practices.

Caesarean delivery has a deleterious effect on the development of the intestinal microbiota in term infants, but it has not been reported in preterm infants [11,12]. In our study, two thirds of the children were born by caesarean section, and we did not find that it was a significant risk factor for MRB.

The composition of intestinal microbiota may influence the development of digestive disorders in children. In our study, most of the VLBW infants had pathogens in stools before the onset of rectal bleeding. We observed *Staphylococcus* in line with the latest data from the literature [11-13]. Although it was the most frequent microorganism apart from *Staphylococcus*, *Clostridium difficile* was rarely found in our study. Our results suggest that *Clostridium difficile* might be the cause of some cases of MRB in VLBW. Furthermore, as the proportion of stool cultures with more than 2 species was less in Cases than in Controls, it could suggest low gut flora diversity in Cases, but nothing can be concluded about stool flora diversity since only pathogens were cultured. Lack of gut flora diversity has been reported as a risk factor for intestinal disorders in preterm infants [14]. However more complete investigation of gut microbiota in case of MRB is required.

According to the findings of Maayan-Metzger et al., only the type of milk was predictive of isolated rectal bleeding. Infants fed milk other than breast milk had four times the risk of MRB (odds ratio = 4.11 [95% CI = 2.41, 6.99]) [5]. It suggest that a possible relationship between rectal bleeding and intolerance to cow’s milk proteins which has been reported in preterm infants [15]. In our study, the type of milk at the time of rectal bleeding did not appear to be a risk factor, probably because feeding practices changed since the previous study performed in infants born between 1996 and 2001 [6]. All infants were fed human milk at least until they reached a body weight of 1500 g. Therefore, as it is presently recommended all infants in our population benefited from protective immunologic factors of human milk for the intestine [16]. We used pasteurized human milk which offers a good combination between microbiological safety and preservation of immunological factors [17]. Therefore, it is the first study evaluating incidence and risk factors of MRB in VLBW infants fed mainly human milk.

Ibuprofen is used in our unit because its efficacy has been shown to be comparable to that of indomethacin for treatment of patent ductus arteriosus, without reducing mesenteric, renal and cerebral blood flows [18,19]. In present study we observed that infants who received ibuprofen had about three times greater chance of having MRB, but it was not designed to determine whether this effect was due to the drug itself or the haemodynamic consequences of persistent ductus arteriosus. Non-steroidal anti-inflammatory drugs are well known to be a predisposing factor for bleeding, but it has not been shown to be a risk factor for rectal bleeding or NEC in preterm infants. Maayan et al. were unable to show such a relationship between non-steroidal anti-inflammatory drugs and rectal bleeding because they studied only low risk babies and excluded infants with special medical treatment including indomethacin [5]. Therefore our study is the first report of ibuprofen being a significant risk factor for MRB in VLBW infants.

Postnatal administration of corticosteroids has been associated with the occurrence of gastrointestinal complications, including intestinal bleeding [20,21]. Nonetheless, postnatal exposure to steroids was not a significant factor of MRB in our population, in neither the univariate nor the multivariate analysis. Dosage, postnatal age at exposure, and associated drugs might be as important as steroid exposure itself.

It has been reported that H2-blocker therapy increases the incidence of necrotizing enterocolitis, suggesting that bacteria play a critical role in the pathogenesis of NEC [22]. Such an effect was not observed in our study, in a population of VLBW in which around one infant out of six received anti-acids, which suggests that the pathogenesis of NEC and MRB are different.

Maternal hypertension is an independent risk factor for the development of NEC in preterm neonates weighing <1500 g [23]. Thus, maternal vascular disorders may play an important role in the pathophysiology of NEC. In our study, hypertension during pregnancy was an independent risk factor for MRB, suggesting that it may induce vascular anomalies significant enough to cause postnatal digestive disorders like rectal bleeding, but not severe enough to have an impact on fetal growth [24,25].

However, our study did not aim to identify the mechanisms underlying or leading to bowel disease. Cow’s milk allergy has been suggested as a possible cause of isolated rectal bleeding related to allergic colitis in term neonates or in older infants [26,27]. In preterm infants the cause of MRB could be different. Severe digestive disorders observed in VLBW infants such as NEC stage ≥ 2 has been related to an inflammatory reaction due to an immunological mechanism related to intestinal intolerance to diet proteins [28,29], or to gut dysmicrobism as in NEC [1,30].

Our results suggest that attention should be paid to a maternal history of hypertension during pregnancy when setting the enteral feeding schedule for VLBW infants. Even if they are not growth restricted at birth, as in our population, perhaps we should consider the history of maternal hypertension and monitor carefully feeding tolerance in these patients. However it is not possible from our data to recommend a specific way to feed these infants and it should be further evaluated in randomized prospective studies.

## Conclusion

In conclusion, in VLBW infants mainly fed human milk, we observed that only hypertension during pregnancy, extra-uterine growth restriction, and postnatal exposure to ibuprofen were significant risk factors for the occurrence of MRB. Therefore, prophylactic measures could be proposed such as careful treatment of hypertension during pregnancy, and a neonatal nutritional care aiming to limit postnatal growth restriction. Prospective studies in larger populations are needed to confirm our results and to evaluate the potential benefits of specific prophylactic measures such as careful nutritional care and rationalized prescription of non-steroidal anti-inflammatory drugs, notably in children of mothers with hypertension during pregnancy.

## Abbreviations

BW: Birth weight; GA: Gestational age; MRB: Mild rectal bleeding; NEC: Necrotising enterocolitis; SD: Standard deviations; VLBW: Very low birth weight.

## Competing interests

The authors (AO, SH, MBS, DMB, IJ, JCP) declare they have no competing interests.

## Authors’ contributions

Conceived and designed the study: AO, SH, MBS, JCP. Performed the study: AO, SH, MBS, IJ, JCP. Analyzed the data: AO, SH, DMB, JCP. Wrote the paper: AO, SH, MBS, DMB, JCP. Participated in data analysis and interpretation: AO, SH, MBS, DMB, IJ, JCP. All authors have read and approved the final manuscript.

## Pre-publication history

The pre-publication history for this paper can be accessed here:

http://www.biomedcentral.com/1471-2431/13/196/prepub
